# Possible role of COVID-19 in the relapse of Klein-Levin Syndrome

**DOI:** 10.1016/j.rmcr.2021.101445

**Published:** 2021-05-29

**Authors:** Adeel Nasrullah, Anam Javed, Obaid Ashraf, Khalid Malik

**Affiliations:** a Department of Medicine, Allegheny Health Network, Pittsburgh, PA, USA; b Departement Pulmonology and Critical Care, Allegheny Health Network, Pittsburgh, PA, USA

**Keywords:** Klein-Levin syndrome, Hyperosmnia, COVID-19

## Abstract

Klein-Levin Syndrome (KLS) is an extremely rare neurological disorder which can manifest as recurring spells of sleepiness, cognitive disturbances and behavioral changes. We present a novel case of KLS relapse in the setting of Coronavirus disease-19 (COVID-19). A 36-year-old male who had a known history of KLS since adolescence was admitted with sleepiness and behavioral disturbances. Brain imaging and autoimmune encephalitis work was unremarkable. The patient was diagnosed with a relapse of KLS secondary to COVID-19 based on symptomology and lack of any other precipitating factor. The patient required 8 days of hospitalization and was treated with benzodiazepines due to a history of robust response to lorazepam during a prior episode. The patient progressively improved and was discharged home on lorazepam taper. We report that similar to other neurotropic viruses, severe acute respiratory syndrome coronavirus 2 (SARS-CoV-2) could be the culprit in instigating KLS relapse.

## Abbreviations

KLS: Klein-Levin Syndrome
COVID-19: Coronavirus disease-19
SARS-CoV-2: Severe acute respiratory syndrome coronavirus −2
IV: Intravenous
CSF: Cerebrospinal fluid
REM: Rapid eye movement
GAD65: Glutamic acid decarboxylase- 65
NMDA: N-methyl-d-aspartate
OSA: Obstructive sleep apnea

## 1 Introduction

Klein-Levin Syndrome (KLS) is a rare disorder characterized by relapsing and remitting episodes of severe hypersomnia accompanied by cognitive and behavioral disturbances [1]. Known triggers include viral illness, substance use, alcohol intake, sleep deprivation, physical and psychological stresses [2]. We report a novel case of KLS relapse possibly secondary to coronavirus disease-19 (COVID-19).

### 1.1 Case presentation

A 36-year-old male with a known history of KLS since age 14 and mild persistent depressive disorder in remission was admitted to the hospital with complaints of confusion, inability to concentrate, and sleepiness lasting over 20 hours per day, and agitation for the past ten days. Two weeks prior the patient had flu-like symptoms and was diagnosed with COVID-19, which was treated conservatively at home. The patient's last known relapse of KLS was five years ago and lasted for 6 weeks but was terminated with intravenous lorazepam over the course of a few days. In the past, the patient had failed prophylactic medications including lithium, valproic acid, trileptal and modafinil. He denied smoking, alcohol or substance use. The patient scored 12/24 on the Epworth sleepiness scale, consistent with abnormal sleepiness, and 10 (normal <3) on the Stanford proxy test of delirium. The patient was noted to have derealization, distress, lability and anxious mood. He had impaired registration, recall, judgement, and lacked insight. Complete blood count, complete metabolic panel, Mayo Clinic serum autoimmune encephalitis panel and MRI brain were unremarkable. The patient was diagnosed with a relapse of KLS secondary to COVID-19. With prior KLS relapses, the patient had failed multiple medications including stimulants, and mood stabilizers. Due to prior robust response to lorazepam the patient was started on Lorazepam 2 mg intravenous (IV) every 4 h for 3 days and Melatonin 10 mg daily. Sleepiness significantly improved and the patient regained capacity by day seven of hospitalization. He received a total of 8 days of lorazepam during hospitalization and was discharged home on 8 days of lorazepam taper with a close follow-up plan with psychiatry. This episode was the patient's shortest KLS relapse.

## 2 Discussion

KLS is typically diagnosed in adolescent males (median age 15), and commonly reported episodic symptoms include hypersomnia, derealization, eating disorders, hypersexuality, compulsions, amnesia and depression (more frequent in women) [[2], [3], [4]]. Each attack usually lasts days to weeks, with asymptomatic periods lasting weeks to months between attacks. KLS is reported in association with influenza virus infection and flu-like illness [2,[5], [6], [7]]. More than one third of COVID-19 patients have experienced sleep disturbances due to disrupted sleep-awake cycles and mood disorders [8]. Although COVID-19 has been seen in association with Guillain-Barre Syndrome, which can lead to rapid eye movement (REM) sleep behavior disorder, our patient represents the first reported case of relapsed KLS possibly due to COVID-19 [9,10]. The exact pathophysiology of KLS is yet to be elucidated. Postulated mechanisms include genetic susceptibility, hypothalamic dysfunction and autoimmune dysfunction [3,11,12]. COVID-19 can result in encephalitis involving temporal lobes, and hippocampus which can precipitate KLS like-symptoms, however brain pathology of KLS patients has been elusive till date [13]. Cerebrospinal fluid (CSF) analysis and electroencephalogram are typically normal and nonspecific. Rare cases showed elevated titers of glutamic acid decarboxylase- 65 (GAD65) anti N-methyl-d-aspartate (NMDA) receptor antibodies in CSF correlating with KLS relapse [4,6]. Brain imaging can be obtained to rule out structural abnormalities; there is also growing evidence of hypoperfusion of frontotemporal areas in KLS patients [14]. Similarly bifrontotemporal hypoperfusion has been reported in COVID-19 cases which can possibly explain KLS symptoms as well [15]. Obstructive sleep apnea (OSA) can coexist with KLS, so a sleep study should be done in high-risk patients as treatment of OSA can result in quick resolution of symptoms [16]. Management strategies vary based on symptomology. Hypersomnia improved in less than 40% cases treated with amphetamines. Depression and behavioral disturbances have shown poor response to antidepressants and neuroleptics, respectively [2]. Among mood stabilizers, lithium is the most effective (41%) in prevention of relapse [2]. In patients with prior episodes lasting longer than 30 days, early institution of IV steroids during relapse (less than 10 days from onset) has been shown to decrease the duration of KLS episodes [17]. Our patient had a poor response to multiple mood stabilizers and stimulants in the past but did respond quickly to benzodiazepines during his last KLS relapse. Hence, we treated our patient with lorazepam which led to significant improvement in a week, contributing to the patient's shortest relapse to date. Usual duration of KLS is 4–6 years with complete recovery and favorable prognosis [18]. The psychological and physical stress as well as social constraints caused by the ongoing pandemic may increase risk of KLS relapse. COVID-19 invasion of CNS can lead to encephalitis which can also manifest in behavioral changes; therefore, with the constellation of symptoms concerning for KLS, a low threshold should be kept to investigate and treat it appropriately [10].

## Prior presentations

None.

## Funding

This research did not receive any specific grant from funding agencies in the public, commercial, or not-for-profit sectors.

## Consent

A written consent to publish this case report has been obtained from the patient. All the authors have seen and approved the manuscript.

## Declaration of competing interest

The authors report no conflicts of interest.
